# Impact of Neonatal Fc Receptor on Transferrin Receptor Antibody Fusion Protein Pharmacokinetics †

**DOI:** 10.3390/pharmaceutics18020269

**Published:** 2026-02-22

**Authors:** Adenike Oyegbesan, Nataraj Jagadeesan, Devaraj V. Chandrashekar, Rachita K. Sumbria

**Affiliations:** 1 Department of Biomedical and Pharmaceutical Sciences, School of Pharmacy, Chapman University, Irvine, CA 92618, USA; oyegbesan@chapman.edu (A.O.); jagadeesan@chapman.edu (N.J.); chandnrashekar@chapman.edu (D.V.C.); 2 Department of Neurology, University of California, Irvine, CA 92697, USA; 3 Center for Targeted Drug Delivery (CTDD), School of Pharmacy, Chapman University, Irvine, CA 92618, USA

**Keywords:** FcRn-mediated recycling, blood-brain barrier, transferrin receptor antibody, neonatal Fc receptor, brain drug delivery

## Abstract

**Background**: Transferrin receptor-targeting monoclonal antibodies (TfRMAbs) enhance brain drug delivery by facilitating TfR-mediated transcytosis across the blood–brain barrier (BBB). Data suggest that chronic TfRMAb dosing reduces their plasma exposure in a dose- and fusion partner-dependent manner; however, the underlying mechanisms remain unclear. The neonatal Fc receptor (FcRn) extends IgG half-life via recycling, but its saturation after repeated doses may alter the pharmacokinetics (PK) of IgG fusion proteins. This study evaluated the role of the FcRn on the PK and biodistribution of TfRMAb fusion proteins. **Methods**: We examined TfRMAb alone and TfRMAb fused to erythropoietin (TfRMAb-EPO) or TNFα receptor (TfRMAb-TNFR) in wild-type (WT) and FcRn knockout (KO) mice following acute (single dose) or chronic (3× weekly for 4 weeks) subcutaneous administration at 3 mg/kg. Plasma levels, tissue biodistribution, and FcRn binding were measured using immunoassays. **Results**: Our results show that fusion partners influenced FcRn-mediated recycling and PK of TfRMAb fusion proteins. After acute dosing, TfRMAb-TNFR exhibited the greatest reduction in plasma exposure in FcRn KO versus WT mice, compared with TfRMAb and TfRMAb-EPO. Chronic dosing reduced the plasma persistence of all fusion proteins in WT mice. In FcRn KO mice, plasma exposure of TfRMAb and TfRMAb-EPO decreased with chronic dosing, whereas TfRMAb-TNFR showed no further reduction. Differences in FcRn binding affinity likely explain these patterns. Tissue distribution largely mirrored plasma concentrations. **Conclusions**: FcRn regulates plasma concentrations of TfRMAb fusion proteins in a fusion partner-dependent manner. While FcRn-mediated protection regulates plasma exposure with acute dosing, additional mechanisms beyond FcRn saturation appear to regulate plasma exposure during chronic dosing.

## 1. Introduction

The blood–brain barrier (BBB) presents a major challenge for the delivery of biologics into the brain, necessitating drug delivery strategies [1]. One of the most widely studied and validated strategies to deliver biologics to the brain utilizes receptor-mediated transcytosis (RMT) receptors that are overexpressed at the BBB, such as transferrin receptor-1 (TfR) [1,2]. Different variants of TfR-targeting antibodies (TfRMAbs) have been developed to facilitate the delivery of large-molecular-weight therapeutic molecules across the BBB [3,4,5,6,7,8,9,10,11]. These antibodies differ in their valency and bind to the receptor with different affinities, sparing the transferrin-binding site of the receptor to keep iron regulation intact [1,2,5].

TfRMAbs fused to different therapeutics are under development for many chronic neurological disorders, including Parkinson’s disease [12] and Alzheimer’s disease [10,13,14,15], and this strategy has been granted regulatory approval in Japan for brain delivery of a therapeutic enzyme for Hunter’s syndrome after a successful phase 3 clinical trial [11,16]. Effective treatment of such chronic diseases requires long-term treatment necessitating continuous dosing. Previous work demonstrated that chronic administration of a 3 mg/kg subcutaneous (SQ) dose of a high-affinity bivalent rat/mouse chimeric TfRMAb for 4 weeks reduced systemic bioavailability compared with a single dose in mice [17]. A similar reduction in systemic bioavailability of TfRMAb was observed following chronic SQ dosing of an isotype control mouse IgG1 [17]. Similarly, chronic intravenous (IV) dosing of a humanized high-affinity bivalent TfRMAb alone for 4 weeks at doses ranging between 3 and 30 mg/kg was associated with a reduction in plasma concentrations in rhesus monkeys [18]. Moreover, chronic IV dosing of a humanized TfRMAb-iduronate-2-sulfatase fusion protein for up to 26 weeks resulted in a reduction in plasma concentrations at doses ranging between 1 and 30 mg/kg in cynomolgus monkeys [19]. On the contrary, chronic dosing of high-affinity bivalent TfRMAb fused to glial-derived neurotrophic factor (GDNF) or a TNF-α inhibitor at lower doses (1–1.75 mg/kg) did not alter plasma pharmacokinetics of the TfRMAb fusion protein in mice [15,20]. The exact mechanisms underlying the reduction in the plasma exposure of TfRMAbs and TfRMAb fusion proteins are not clearly defined but include the formation of anti-drug antibody (ADA), an increase in peripheral TfR expression, fusion partner-specific clearance mechanisms, and saturation of the neonatal fragment crystallizable receptor (FcRn) [17,19,21,22]; the latter is the focus of this study.

Development of ADAs can accelerate clearance of monoclonal antibodies through immune complex formation and subsequent removal by Fc gamma receptor (FcγR)-expressing cells, particularly within the liver and spleen [23]. This process is further influenced by adaptive immune responses, as CD4^+^ T cells play a critical role in T cell-dependent ADA formation, affinity maturation, and isotype switching, thereby modulating antibody persistence and clearance [24]; these processes have been implicated in enhanced TfRMAb fusion protein clearance [21,22]. Furthermore, increased peripheral TfR expression may enhance receptor-mediated uptake of TfRMAb-based fusion proteins in non-target tissues [17], leading to accelerated clearance. Additionally, binding of the TfRMAb fusion partner to specific receptors may induce receptor-mediated internalization and lysosomal degradation, impacting TfRMAb fusion protein clearance [22]. This receptor-mediated clearance constitutes target-mediated drug disposition and has been shown to contribute to non-linear clearance of monoclonal antibodies [3,25].

FcRn plays a crucial role in maintaining the long circulating half-life of IgGs. This process of FcRn-mediated recycling involves binding of the IgG Fc domain to the FcRn at an acidic pH, lysosomal escape of the IgG, and IgG release into the circulation at physiological pH [26]. Accordingly, strategies to enhance FcRn binding of therapeutic antibodies through Fc domain engineering have significantly improved antibody recycling and extended half-life, thereby possibly increasing their therapeutic efficacy [27]. Conversely, engineering antibody-binding fragment (Fab) antibody fusion proteins without the Fc domain is a widely used strategy to reduce Fc-mediated effector function side effects but results in decreased systemic exposure and shorter duration of action [27,28]. This reduction in systemic exposure is also observed with long-term antibody dosing, which can potentially saturate FcRn-mediated recycling and increase antibody catabolism [27].

Although the role of FcRn in regulating the systemic half-life of IgG and Fc fusion proteins is well established, studies examining the FcRn-mediated rescue of TfRMAbs have been limited. A recent study investigated the role of FcRn in mediating the systemic and brain exposure of a TfRMAb Fab fragment fused to an anti-amyloid antibody [29]. A significant increase in systemic clearance was observed in the presence of a mutation in the FcRn binding domain of the anti-amyloid antibody after a single IV injection [29]. However, the contribution of FcRn in regulating plasma and brain concentrations of full-length TfRMAb and associated fusion proteins after extravascular chronic dosing, which is the preferred route of administration to treat chronic diseases, is unclear. Therefore, the aim of the current study was two-fold: to elucidate the role of FcRn in regulating acute and chronic plasma pharmacokinetics and biodistribution of a high-affinity bivalent full-length mouse/rat chimeric TfRMAb [30]; and to determine if FcRn-mediated rescue is a function of the therapeutic fusion partner attached to the TfRMAb. For this, we used TfRMAb and two model TfRMAb fusion proteins, TfRMAb fused to a neurotrophin (erythropoietin) (TfRMAb-EPO) and TfRMAb fused to the extracellular domain of the type II TNF-α receptor (TfRMAb-TNFR), and examined how FcRn regulates systemic exposure and biodistribution following acute and chronic dosing in wild-type (WT) and FcRn knockout (KO) mice. The results of the study demonstrate a significant, albeit variable, role of FcRn in sustaining systemic and tissue exposure of TfRMAb fusion proteins. In addition, the findings provide insights into how the FcRn regulates the systemic and tissue persistence of TfRMAb therapeutics following single and repeated dosing. These observations may also be useful when considering the engineering of TfRMAb Fab fusion proteins, which are currently under development [31].

## 2. Materials and Methods

### 2.1. Transferrin Receptor Monoclonal Antibody Fusion Proteins

The TfRMAb-TNFR and TfRMAb-EPO fusion proteins were produced via transient expression in Chinese hamster ovary (CHO-K1) cells [32,33]. Following expression, the fusion proteins were purified using protein A affinity chromatography and size-exclusion chromatography to ensure high purity. The purified TfRMAb-TNFR and TfRMAb-EPO fusion proteins were formulated at concentrations of 1.1 mg/mL and 1.49 mg/mL, respectively. TfRMAb-TNFR was prepared in a buffer containing 0.01 M sodium acetate, 0.148 M NaCl, and 0.01% polysorbate 80 at pH 5.5, while TfRMAb-EPO was formulated in a buffer containing 0.05 M sodium acetate, 0.148 M NaCl, and 0.01% polysorbate 80 at pH 5.5. The solutions were sterile-filtered and stored at −80 °C until needed. To produce TfRMAb, ExpiCHO cells were cultured in serum-free expression medium. The expressed protein was formulated at a concentration of 1.05 mg/mL in 10 mM sodium acetate, 150 mM NaCl, and 0.01% polysorbate 80 at pH 6.0 [17]. The TfRMAb protein was sterile-filtered and stored at −80 °C for future use. The TfRMAb used herein is derived from the rat monoclonal TfRMAb, 8D3, which is the most widely used TfRMAb targeting the mouse TfR for brain drug delivery [34]. However, to reduce immune reaction in the mouse, a chimeric TfRMAb was engineered by fusing the variable heavy and light chains of the 8D3 antibody to the constant region of the mouse IgG1 heavy chain and the constant region of the mouse kappa light chain [30]. This yields a rat/mouse chimeric TfRMAb suitable for use in a mouse without interfering with transferrin binding to TfR.

### 2.2. Acute Dosing of TfRMAb Fusion Proteins

All experimental procedures were conducted in accordance with the protocol (2010-1170) approved by the Chapman University Institutional Animal Care and Use Committee. The study utilized 8-week-old male wild-type (WT, C57BL/6 JAX Stock Number: 000664) and male FcRn KO mice (B6.129X1-Fcgrt<tm1Dcr>/DcrJ JAX Stock Number: 003982) purchased from The Jackson Laboratory (Bar Harbor, ME, USA), which were housed under standard conditions with unrestricted access to food and water and maintained on a 12 h light/dark cycle. Mice were randomized into treatment groups based on body weight (average mouse weights at the beginning of the study: WT: 22.9 ± 0.89 g, FcRn KO: 22.2 ± 1.5 g) to ensure comparable group averages at the start of the study. The treatment groups received SQ injections of TfRMAb (3 mg/kg, *n* = 5), TfRMAb-TNFR (3 mg/kg, *n* = 5), or TfRMAb-EPO (3 mg/kg, *n* = 5). Following injections, mice were closely monitored for 2 h to assess their general appearance and posture to rule out any severe immune response [17].

For blood collection, mice were briefly anesthetized using 2% isoflurane (Vetone Fluriso, 502017, MWI Animal Health, Boise, ID, USA) delivered using the Somnoflo anesthesia system (Kent Scientific, KENT-SS-0, Torrington, CT, USA), and blood samples were collected in sodium citrate (Thermo Fisher Scientific, 02-683-172, Waltham, MA, USA) as 9 parts blood 1 part sodium citrate from the retro-orbital sinus at 3, 6, and 24 h post-injection. These sampling times were selected based on previous results showing a Tmax between 3 and 6 h for TfRMAb-TNFR, TfRMAb, and TfRMAb-EPO [17,33,35]. Plasma was collected by centrifuging blood samples at 10,000× *g* for 5 min at 4 °C, aliquoted, and stored at −80 °C to prevent multiple freeze–thaw cycles. Plasma was analyzed by immunoassays to quantify plasma fusion protein concentrations. At the terminal 24 h timepoint, mice were injected with Euthasol intraperitoneally (150 mg/kg), and while anesthetized, immediately perfused transcardially with ice-cold phosphate-buffered saline (PBS) to clear the blood vessels. Perfusion efficiency was confirmed by clear perfusate and blanching of highly vascular tissues, and organs, including the brain, liver, kidney, and spleen, were harvested. Tissues were snap-frozen in liquid nitrogen to analyze fusion protein concentrations. Additional blood samples were collected in lithium-heparin tubes (SARSTEDT Inc., 41.1393.105, Newton, NC, USA), and plasma was separated by centrifugation. Terminal plasma samples were used for a comprehensive diagnostic panel using a VetScan rotor (Abaxis, 10023220, Union City, CA, USA) and a VetScan VS2 chemical analyzer (Abaxis, 1200-1000, Union City, CA, USA).

### 2.3. Chronic Dosing of TfRMAb Fusion Proteins

All animal studies were conducted under protocol 2010-1170, approved by the Chapman University Institutional Animal Care and Use Committee. The study utilized 8-week-old male WT and FcRn KO mice (Jackson Laboratory, Bar Harbor, ME, USA). Mice were housed under standard conditions with ad libitum access to food and water and maintained on a 12 h light/dark cycle. Mice were randomized into treatment groups based on body weight to ensure similar group averages before the study start (average mouse weights at the beginning of the study: WT: 21.1 ± 1.2 g, FcRn KO: 21.7 ± 0.85 g). Mice were injected SQ with TfRMAb, TfRMAb-TNFR, or TfRMAb-EPO fusion proteins at a dosage of 3 mg/kg, three times a week for four weeks. Each treatment group consisted of five mice. Mice were monitored carefully after injection, and a complete health assessment was performed weekly after injection for general appearance [17]. After the final injection, blood samples were collected at 3, 6, and 24 h post-injection, and plasma was isolated and stored as described below. Briefly, blood samples were collected via the retro-orbital sinus under 2% isoflurane anesthesia (Vetone Fluriso, 502017, MWI Animal Health, Boise, ID, USA) delivered using the Somnoflo anesthesia system (Kent Scientific, KENT-SS-0, Torrington, CT, USA) into sodium citrate-containing tubes (Thermo Fisher Scientific, 02-683-172, Waltham, MA, USA). Plasma was separated by centrifuging the blood samples at 10,000× *g* for 5 min at 4 °C. Plasma aliquots were frozen and stored in well-labeled Eppendorf vials at −80 °C until immunoassays to determine fusion protein concentrations. At the terminal time-point, mice were injected with Euthasol intraperitoneally (150 mg/kg), and while anesthetized, immediately perfused transcardially with ice-cold PBS to clear the blood vessels. Perfusion efficiency was confirmed by clear perfusate and blanching of highly vascular tissues, and organs, including the brain, liver, kidney, and spleen, were harvested. Tissues were gently blotted on filter paper and snap-frozen in liquid nitrogen. Hematology analysis was performed on terminal whole-blood samples collected in Microvette potassium-EDTA tubes (SARSTEDT Inc., 20.1278.100, Newton, NC, USA) at 4 °C, either on the day of collection or within 24 h, using the VetScan HM5 hematology analyzer (Zoetis, 10023319, Parsippany, NJ, USA).

### 2.4. Plasma TfRMAb and TfRMAb Fusion Protein Concentrations by ELISA

Plasma concentrations of TfRMAb, TfRMAb-TNFR, and TfRMAb-EPO were determined using a sandwich ELISA [17,33]. Nunc Maxisorp plates (Fisher Scientific, 501123685, Hanover Park, IL, USA) were coated overnight at 4 °C with recombinant human EPOR/Fc fusion protein (200 ng/well) to measure TfRMAb-EPO (R&D systems, 963-ER, Minneapolis, MN, USA) or recombinant murine TfR (200 ng/well) to measure TfRMAb and TfRMAb-TNFR (R&D systems, 9706-TR, Minneapolis, MN, USA) in 0.1 M or 0.05 M NaHCO3, pH 8.3 (Sigma-Aldrich, S5761, St. Louis, MO, USA), respectively. Following overnight incubation, the coating solution was aspirated, and the wells were washed three times with Tris-buffered saline containing 0.05% Tween-20 (Bio-rad Laboratories, 1200274186, Hercules, CA, USA) (TBST). Plates were blocked with TBS containing 1% bovine serum albumin (BSA) (Roche Diagnostics GmbH, 10735078001, Mannheim, Germany) (TBSB) for 30 min at room temperature (RT) to prevent non-specific binding. After blocking, 100 μL of the appropriate standards or diluted plasma samples (1:10 in TBSB) for TfRMAb, TfRMAb-TNFR, and TfRMAb-EPO were added to the wells and incubated for up to 2 h at RT. Plates were washed three times with TBST to remove unbound proteins. For TfRMAb-TNFR measurements, an additional step included incubation with rabbit anti-TNFRII (Sino Biologicals, 20230119980430, Paoli, PA, USA) for 30 min at RT after removing unbound proteins. Goat anti-mouse alkaline phosphatase conjugate (100 ng/well) (Bethyl Laboratories Inc., A90-119A, Montgomery, TX, USA) that binds to the TfRMAb domain was added and incubated for 45 min at RT to measure TfRMAb and TfRMAb-EPO. Goat anti-rabbit IgG alkaline phosphatase conjugate (100 ng/well) (Invitrogen, 656122, Waltham, MA, USA) that binds to the anti-TNFRII was added and incubated for 45 min at RT to measure TfRMAb-TNFR. Plates were then washed three more times with TBST and incubated with p-nitrophenyl phosphate substrate (Sigma-Aldrich, P5994, St. Louis, MO, USA). The substrate was incubated in the dark for 5–15 min to allow for color development, with careful observation to prevent overdevelopment. The reaction was stopped by adding 100 μL of 1.2 M NaOH per well, and absorbance was measured at 405 nm using a plate reader. Blank-corrected absorbance values were used to calculate plasma concentrations of TfRMAb, TfRMAb-TNFR, and TfRMAb-EPO. The accuracy and detectable concentration range of the TfRMAb ELISAs were established by back-calculated recovery of known standards compared with calculated concentrations across assay runs. The detectable plasma concentration range was 3–900 ng/mL, within which mean ± SD % recovery was 108.7% ± 17.2, meeting standard bioanalytical acceptance criteria (80–120%). Samples outside this range were diluted further or reported as the lowest detectable value (3 ng/mL). Plasma concentrations at 3, 6, and 24 h were reported in ng/mL and were used to calculate the area under the curve (AUC) from 0 to 24 h using the trapezoid rule. These sampling times were selected based on previous results showing a Tmax between 3 and 6 h for TfRMAb-TNFR, TfRMAb, and TfRMAb-EPO [17,33,35]. We acknowledge that late-phase concentration dynamics between 6 h and 24 h may be under-sampled with this design.

### 2.5. Tissue Processing

Frozen organs (spleen, kidney, liver, and brain) were pulverized into a fine powder using an ice-cold pulverizer in a cold room. Approximately 40 mg of spleen, 80 mg of kidney and liver, and 50 mg of brain tissue were weighed and homogenized in ice-cold Tissue Protein Extraction Reagent (TPER) buffer (ThermoScientific, 78510, Waltham, MA, USA) containing EDTA-free protease inhibitors (Sigma, 3032642568, St. Louis, MO, USA) and Halt™ Phosphatase Inhibitor Single-Use Cocktail (ThermoScientific, P178420, Waltham, MA, USA) on ice. Homogenization of spleen, kidney, and liver was performed using 5 µL of buffer per mg of tissue, while brain homogenization was performed using 2 µL of buffer per mg of tissue. Homogenates were rotated at 4 °C for 1 h, followed by centrifugation at 14,000× *g* for 20 min at 4 °C. Supernatants were aliquoted into labeled Eppendorf vials and frozen at −80 °C until further analysis.

### 2.6. Mesoscale Discovery Electrochemiluminescence Assay

A mesoscale discovery (MSD) electrochemiluminescence assay was used to determine the concentration of TfRMAb and TfRMAb fusion proteins in tissue homogenates [22]. MSD GOLD™ 96-well Small Spot Streptavidin SECTOR 96-well plates (Meso Scale Diagnostics, L45SA, Rockville, MD, USA) were blocked with TBS with 3% BSA (Roche Diagnostics GmbH, 10735078001, Mannheim, Germany) and incubated at RT for 1 h at 900 rpm shaking. The plates were incubated with biotinylated EPOR (BPS Bioscience, 100612, San Diego, CA, USA) to enable capture of TfRMAb-EPO, biotinylated TNFα (Sino Biological, 10602-H49H, Paoli, PA, USA) to capture TfRMAb-TNFR, and biotinylated mouse TfR (ACROBiosystems, TFR-M8249, Newark, DE, USA) to capture TfRMAb, at 0.25 µg/mL in TBST with 1% BSA (Roche Diagnostics GmbH, 10735078001, Mannheim, Germany) (TBSB) for 1 h at RT with shaking (900 rpm), followed by three washes with TBST. After the addition of the tissue homogenates and standards to the wells, the plate was incubated for 1 h at RT with shaking (900 rpm). Goat anti-mouse sulfo-tag antibody (Meso Scale Diagnostics, R32AC-5, Rockville, MD, USA) (1 µg/mL in 1% BSA in TBS at 25 ng/well) was added to each well. After 1 h incubation at RT with shaking (900 rpm), plates were washed three times with TBST and Gold Read Buffer B (Meso Scale Diagnostics, B-R60AM-3, Rockville, MD, USA) was added, and signals were read using an MSD MESO QuickPlex SQ 120 96-well plate reader (Meso Scale Diagnostics, AI0AA-0, Rockville, MD, USA) [22]. Recovery in brain homogenates was tested at three spiked concentrations: spleen spike range: 0.1–100 ng/mL; kidney spike range: 0.15–15 ng/mL; liver spike range: 0.5–50 ng/mL; and brain spike range: 0.03–3 ng/mL. The average recovery across the three spiked concentrations for the different organs was as follows for the mean ± SEM: brain: 104 ± 18, liver: 117 ± 6, and kidney: 100 ± 12. For the spleen, a significant matrix effect was seen at spike concentrations of 0.1 ng/mL, but the average recovery for higher spike concentrations was 116 ± 35.

### 2.7. FcRn Binding of Mouse IgG, TfRMAb, and TfRMAb Fusion Proteins

The binding affinity to FcRn was determined via an electrochemiluminescence-based assay using the MSD technology and MSD GOLD™ Small Spot Streptavidin plates (Meso Scale Diagnostics, L45SA, Rockville, MD, USA) described above. Prior to use, wells were blocked with PBS (pH 6.0) containing 3% BSA (Roche Diagnostics GmbH, 10735078001, Mannheim, Germany), followed by a 1 h incubation at RT under constant shaking (900 rpm). After blocking, 30 µL/well of biotinylated mouse FcRn (Kactus Bio, FRN-MM401B, Waltham, MA, USA) diluted to 0.2 µg/well in PBS containing 1% BSA (Roche Diagnostics GmbH, 10735078001, Mannheim, Germany) (PBSB, pH 6.0) was added, with select wells left uncoated as negative controls. The plate was incubated for 1 h at RT with shaking, followed by three washes with 150 µL/well PBS containing 0.05% Tween-20 (Bio-rad Laboratories, 1200274186, Hercules, CA, USA) (PBST, pH 6.0). Mouse IgG1, TfRMAb, TfRMAb-EPO, and TfRMAb-TNFR were prepared in PBSB pH 6.0 by serial dilution of stock solutions (TfRMAb: 1.05 mg/mL; TfRMAb-EPO: 1.49 mg/mL; TfRMAb-TNFR: 1.1 mg/mL; mouse IgG1: 9.78 mg/mL). For TfRMAb, mouse IgG1, and TfRMAb-EPO, standards spanned 200,000–78.13 ng/mL (5000–1.95 ng/well in 25 µL). For TfRMAb-TNFR, standards ranged from 800,000 to 78.13 ng/mL (20,000–1.95 ng/well). Standards (25 µL/well) were added in duplicates and incubated for 1 h at RT with shaking. After three PBST washes, sulfo-tagged detection Fab antibody was added, which was prepared by conjugating the Fab fragment (Jackson Immunoresearch, 115-006-072, West Grove, PA, USA) with Sulfo-TAG NHS-Ester (Meso Scale Diagnostics, R91AN-1, Rockville, MD, USA) using a 20:1 molar challenge ratio. The use of Fab antibody is important to prevent non-specific binding to the coated biotinylated mouse FcRn. Briefly, 7.3 µL of reconstituted Sulfo-TAG reagent (3 nmol/µL in distilled water) was added to 100 µL of Fab antibody (1.3 mg/mL) and incubated at RT for 2 h (protected from light). The conjugated Fab was diluted to 25 ng/well in PBS containing 1% BSA (Roche Diagnostics GmbH, 10735078001, Mannheim, Germany) (pH 6.0), added to wells (25 µL/well), and incubated for 1 h at RT with shaking. Following three PBST washes, 150 µL/well of Gold Read Buffer B (Meso Scale Diagnostics, B-R60AM-3, Rockville, MD, USA) was added, and the plate was immediately analyzed using an MSD MESO QuickPlex SQ 120 96-well plate reader (Meso Scale Diagnostics, AI0AA-0, Rockville, MD, USA). The data was collected and analyzed.

### 2.8. Statistical Analysis

The data are presented as the mean ± SEM, and statistical analyses were conducted using GraphPad Prism (v10.04.1, La Jolla, CA, USA). Outliers were identified and removed using Grubb’s outlier test. Using G*Power version 3.1.9.7 for sample size estimation, it was determined that 3–5 mice per group would be sufficient to detect an effect size of 35–50%, with a standard deviation of 15%, 80% power, and a significance level of 5%, as supported by previous studies [22,33]. A one-way ANOVA test followed by Holm–Sidak post hoc test were used for comparing more than two independent groups. A repeated-measures two-way ANOVA test was used to evaluate the plasma drug concentrations over time in WT and FcRn KO mice, followed by the Holm–Sidak post hoc analysis. Significance level for all the tests was set to 0.05, and statistical significance was established at a two-tailed *p* ≤ 0.05.

## 3. Results

### 3.1. Plasma Pharmacokinetics and Tissue Biodistribution of TfRMAb-EPO Following Acute and Chronic Dosing

**Figure 1:**
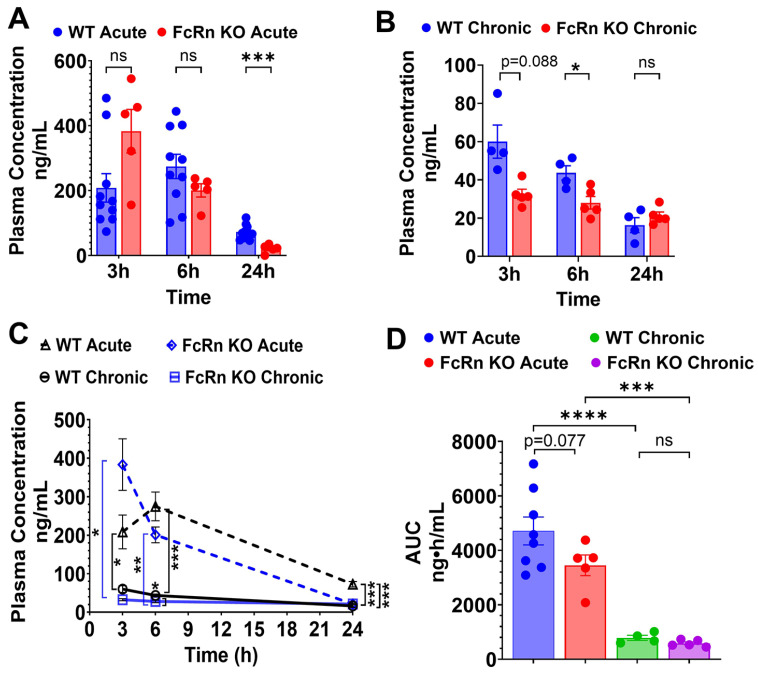
Plasma pharmacokinetics of TfRMAb-EPO following acute and chronic dosing. WT and FcRn KO mice received acute or chronic four-week dosing of TfRMAb-EPO (3 mg/kg SQ). (**A**) Plasma concentrations (ng/mL) at 3 h, 6 h, and 24 h after acute dosing and (**B**) after one final dose following chronic dosing. (**C**) Merged plasma concentration vs. time curves for acute and chronic dosing regimens. (**D**) Plasma AUC from 0 to 24 h (ng·h/mL) following acute and chronic dosing. Data are shown as the mean ± SEM of *n* = 4–10 mice per group. * *p* < 0.05; ** *p* < 0.01; *** *p* < 0.001; **** *p* < 0.0001; ns: non-significant.

**Figure 2:**
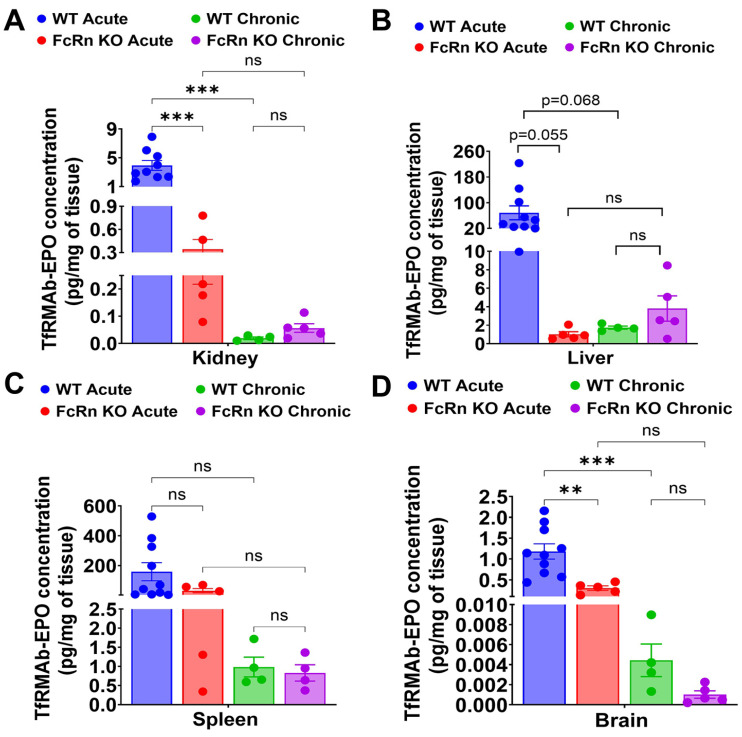
Tissue biodistribution of TfRMAb-EPO following acute and chronic dosing. Biodistribution of TfRMAb-EPO following a single 3 mg/kg SQ dose (acute) or four weeks of chronic dosing. Tissue concentrations (pg/mg of tissue) were measured in (**A**) kidney, (**B**) liver, (**C**) spleen, and (**D**) brain. Data are shown as the mean ± SEM of *n* = 4–10 mice per group. ** *p* < 0.01; *** *p* < 0.001; ns: non-significant.

The plasma concentrations of TfRMAb-EPO were evaluated following a single 3 mg/kg SQ dose (Figure 1). Following dosing to WT mice, plasma concentrations of TfRMAb-EPO declined from 208.4 ± 43.9 ng/mL at 3 h to 72.8 ± 7.10 ng/mL at 24 h (Figure 1A,C). In the FcRn KO mice, plasma concentrations of TfRMAb-EPO declined from 383.3 ± 67.2 ng/mL at 3 h to 21.2 ± 5.9 ng/mL at 24 h (Figure 1A,C). The plasma concentrations of TfRMAb-EPO were significantly higher in the WT mice than in FcRn KO mice at 24 h (72.8 ± 7.10 ng/mL vs. 21.2 ± 5.9 ng/mL, *p* < 0.001) (Figure 1A,C), showing the impact of the loss of FcRn-mediated recycling on TfRMAb-EPO concentrations at later time points after a single SQ injection. The decline in plasma concentration from 3 h to 24 h was also significantly greater in FcRn KO mice (362.2 ± 152.1 ng/mL) compared with WT mice (135.7 ± 124.5 ng/mL, *p* < 0.01), indicating faster clearance of the TfRMAb-EPO in FcRn KO mice. The plasma area under the curve (AUC) from 0 to 24 h following a single SQ dose of TfRMAb-EPO was approximately 32% lower in FcRn KO mice than in WT mice, though this difference was not statistically significant (*p* = 0.077) (Figure 1D). Tissue biodistribution analysis (Figure 2A–D) following acute dosing revealed that the concentration of TfRMAb-EPO in the FcRn KO mice was significantly lower in the kidney and brain compared with WT mice by 87.7% (*p* < 0.001) and 74.6% (*p* < 0.01), respectively (Figure 2A,D). The same trend was observed in the liver and spleen, where the concentration of TfRMAb-EPO in the FcRn KO mice was lower by 98.5% (*p* = 0.055) and 80.3% (*p* = 0.24), respectively, though this difference was not statistically significant (Figure 2B,C).

Following four weeks of chronic dosing with a 3 mg/kg SQ dose, mice were injected with a final 3 mg/kg SQ dose of TfRMAb-EPO, and plasma concentrations were measured at 3, 6, and 24 h after this terminal injection. In the WT mice, the plasma concentrations of TfRMAb-EPO declined from 59.6 ± 7.8 ng/mL at 3 h to 16.3 ± 3.5 ng/mL at 24 h (Figure 1B,C). In the FcRn KO mice, plasma concentrations of TfRMAb-EPO declined from 32.4 ± 2.7 ng/mL at 3 h to 21.2 ± 1.9 ng/mL at 24 h (Figure 1B,C). The plasma concentrations of TfRMAb-EPO were higher in WT mice than in the FcRn KO mice at 3 h (*p* = 0.088) and 6 h after the last injection, with the difference being statistically significant at 6 h (43.6 ± 3.4 ng/mL in the WT mice vs. 28.1 ± 7.3 ng/mL in the FcRn KO mice; *p* < 0.05) (Figure 1B,C). The plasma AUC from 0 to 24 h following the last SQ dose of TfRMAb-EPO was approximately 26% lower in FcRn KO mice than in WT mice, and this difference was not statistically significant (Figure 1D).

A comparison of acute and chronic dosing showed that chronic dosing resulted in a significant reduction in the plasma concentrations of TfRMAb-EPO compared with a single injection, both in the WT and FcRn KO mice (Figure 1C). In chronically dosed WT mice, plasma concentrations of TfRMAb-EPO were significantly lower at 3, 6, and 24 h after injection compared with acute dosing (*p* < 0.05, *p* < 0.001, and *p* < 0.001, respectively) (Figure 1C). Similarly, acutely dosed FcRn KO mice had higher plasma concentrations at 3 h and 6 h compared with chronically dosed FcRn KO mice (*p* < 0.05 and *p* < 0.01, respectively), but not at 24 h after the terminal injection (Figure 1C). Chronic dosing of TfRMAb-EPO resulted in a profound 83% reduction (*p* < 0.001) in plasma AUC values in both the FcRn KO and WT mice (Figure 1D). With respect to tissue biodistribution, paralleling a reduction in plasma concentrations in the WT mice, chronic dosing resulted in a significant decrease in TfRMAb-EPO tissue levels in the kidney and brain by 99.5% (*p* < 0.001) and 99.6% (*p* < 0.001), respectively, compared with concentrations following acute dosing (Figure 2A,D). The same trend was observed in the liver and spleen, and chronic dosing reduced TfRMAb-EPO tissue levels of WT mice by 97.5% (*p* = 0.068) and 99.4% (*p* = 0.19), respectively, compared with acute dosing; however, this decrease was not statistically significant (Figure 2B,C). In the FcRn KO mice, chronic dosing of TfRMAb-EPO showed a trend toward a decrease in tissue levels in the kidney, spleen, and brain by 83.5% (*p* = 0.93), 95.2% (*p* = 0.92), and 99.7% (*p* = 0.43), respectively, compared with FcRn KO mice that received a single dose of TfRMAb-EPO (Figure 2A,C,D). In the livers of FcRn KO mice, the reverse was seen, where chronic dosing of TfRMAb-EPO led to an increase in TfRMAb-EPO levels, although this change was not statistically significant (Figure 2B).

### 3.2. Plasma Pharmacokinetics and Tissue Biodistribution of TfRMAb Following Acute and Chronic Dosing

**Figure 3:**
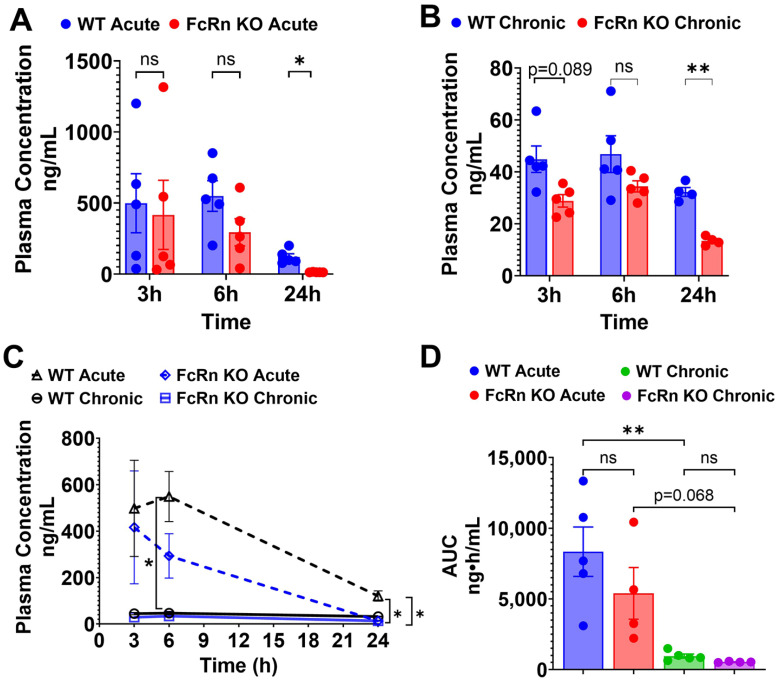
Plasma pharmacokinetics of TfRMAb following acute and chronic dosing. WT and FcRn KO mice received acute or chronic four-week dosing of TfRMAb (3 mg/kg SQ). (**A**) Plasma concentrations (ng/mL) at 3 h, 6 h, and 24 h after acute dosing and (**B**) after one final dose following chronic dosing. (**C**) Merged plasma concentration vs. time curves for acute and chronic dosing regimens. (**D**) Plasma AUC from 0 to 24 h (ng·h/mL) following acute and chronic dosing. Data are shown as the mean ± SEM of *n* = 4–5 mice per group. * *p* < 0.05; ** *p* < 0.01. ns: non-significant.

**Figure 4:**
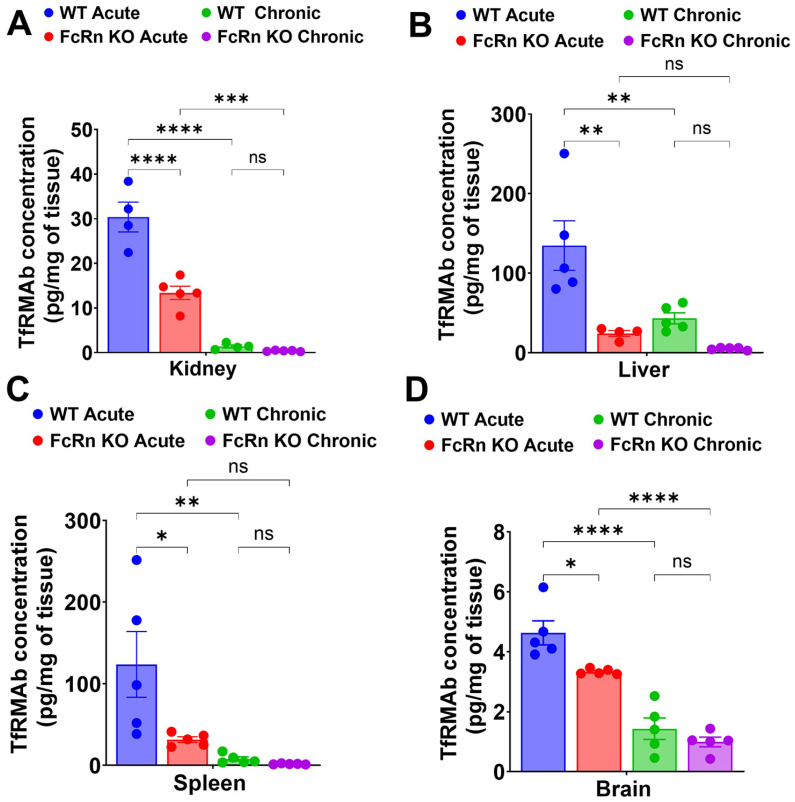
Tissue biodistribution of TfRMAb following acute and chronic dosing. Biodistribution of TfRMAb following a single 3 mg/kg SQ dose (acute) or four weeks of chronic dosing. Tissue concentrations (pg/mg of tissue) were measured in (**A**) kidney, (**B**) liver, (**C**) spleen, and (**D**) brain. Data are shown as the mean ± SEM of *n* = 4–5 mice per group. * *p* < 0.05; ** *p* < 0.01; *** *p* < 0.001; **** *p* < 0.0001. ns: non-significant.

Following single acute SQ dosing to WT mice, plasma concentrations of TfRMAb declined by 76% from 497.9 ± 207.3 ng/mL at 3 h to 120.5 ± 22.3 ng/mL at 24 h (Figure 3A,C). In the FcRn KO mice, plasma concentrations of TfRMAb declined 97% from 416.1 ± 243.1 ng/mL at 3 h to 12.9 ± 0.7 ng/mL at 24 h (Figure 3A,C). The plasma concentrations of TfRMAb were significantly higher in the WT mice than in FcRn KO mice at 24 h (120.5 ± 22.3 ng/mL vs. 12.9 ± 0.7 ng/mL, *p* < 0.05) (Figure 3A,C), showing the impact of the loss of FcRn-mediated recycling on TfRMAb concentrations at later time points after a single SQ injection. The plasma AUC from 0 to 24 h following a single SQ dose of TfRMAb was approximately 35% lower in FcRn KO mice than in WT mice, but this difference was not statistically significant (Figure 3D). Tissue biodistribution analysis following acute dosing revealed that the concentration of TfRMAb in the FcRn KO mice was significantly lower in the kidney, liver, spleen, and brain compared with WT mice by 66.2% (*p* < 0.0001), 74.7% (*p* < 0.01), 74.6% (*p* < 0.05), and 27.8% (*p* < 0.05), respectively, (Figure 4A–D).

Following four weeks of chronic TfRMAb dosing with a 3 mg/kg SQ dose, mice were injected with a final 3 mg/kg SQ dose of TfRMAb, and plasma concentrations were measured at 3, 6, and 24 h after this terminal injection. In the WT mice, the plasma concentrations of TfRMAb declined from 44.9 ± 5.1 ng/mL at 3 h to 32.3 ± 1.5 ng/mL at 24 h (Figure 3B,C). In the FcRn KO mice, plasma concentrations of TfRMAb declined from 28.8 ± 2.4 ng/mL at 3 h to 13.4 ± 0.75 ng/mL at 24 h (Figure 3B,C). The plasma concentrations of TfRMAb showed a trend toward being higher at 3 h (*p* = 0.089) and were significantly higher at 24 h (*p* < 0.01) after the last injection in WT mice than in the FcRn KO mice (Figure 3B,C). The plasma AUC from 0 to 24 h following the last SQ dose of TfRMAb did not differ significantly between the FcRn KO mice and WT mice (Figure 3D). No difference was observed in the TfRMAb levels in the kidney, liver, spleen, and brain between the chronically dosed WT and FcRn KO mice (Figure 4A–D).

A comparison of acute and chronic dosing showed that chronic dosing resulted in a significant reduction in the plasma exposure of TfRMAb compared with a single injection, both in the WT and FcRn KO mice (Figure 3C,D). Chronic dosing of TfRMAb resulted in a profound 88% reduction (*p* < 0.01) and 90% reduction (*p* = 0.068) in plasma AUC values in the WT mice and FcRn KO mice, respectively (Figure 3D), compared with a single dose.

With respect to tissue biodistribution, paralleling a reduction in plasma concentrations in the WT mice, chronic dosing resulted in a significant decrease in TfRMAb tissue levels in the kidney, liver, spleen, and brain by 93.5% (*p* < 0.0001), 68% (*p* < 0.01), 93.7% (*p* < 0.01), and 69% (*p* < 0.0001), respectively, compared with concentrations following acute dosing (Figure 4A–D). In the FcRn KO mice, chronic dosing of TfRMAb significantly reduced the tissue levels in the kidney (*p* < 0.001) and brain (*p* < 0.0001), with a similar trend in the spleen and liver (Figure 4A–D).

### 3.3. Plasma Pharmacokinetics and Tissue Biodistribution of TfRMAb-TNFR Following Acute and Chronic Dosing

**Figure 5:**
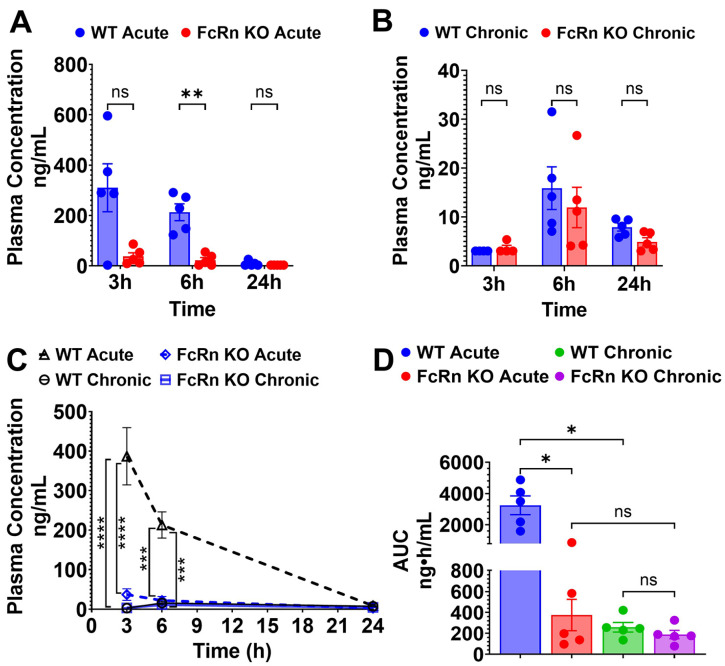
Plasma pharmacokinetics of TfRMAb-TNFR following acute and chronic dosing. WT and FcRn KO mice received acute or chronic four-week dosing of TfRMAb-TNFR (3 mg/kg SQ). (**A**) Plasma concentrations (ng/mL) at 3 h, 6 h, and 24 h after acute dosing and (**B**) after one final dose following chronic dosing. (**C**) Merged plasma concentration vs. time curves for acute and chronic dosing regimens. (**D**) Plasma AUC from 0 to 24 h (ng·h/mL) following acute and chronic dosing. Data are shown as the mean ± SEM of *n* = 4–5 mice per group. * *p* < 0.05; ** *p* < 0.01; *** *p* < 0.001; **** *p* < 0.0001. ns: non-significant.

**Figure 6:**
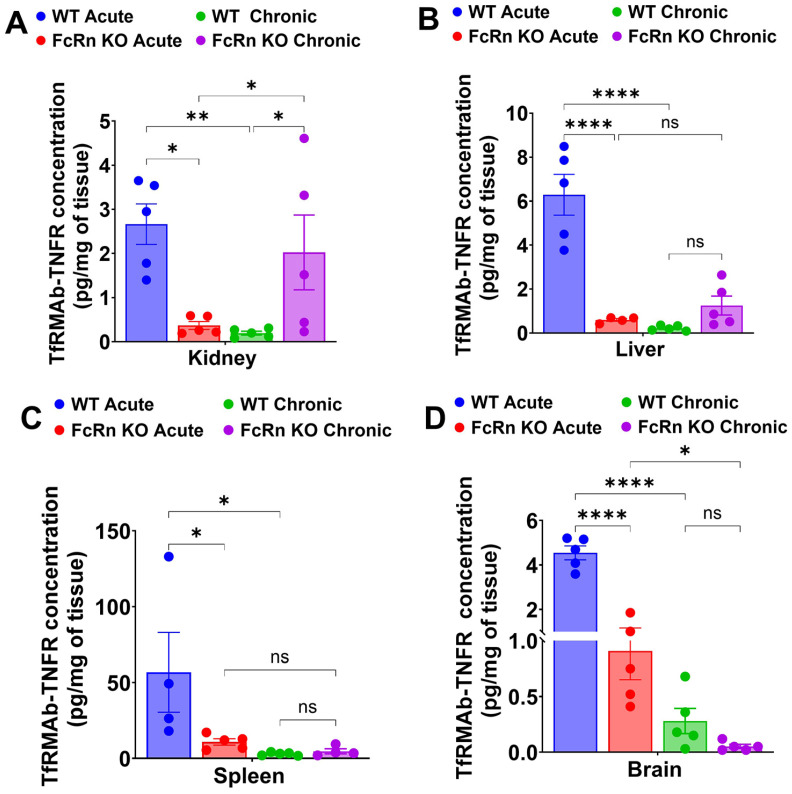
Tissue biodistribution of TfRMAb-TNFR following acute and chronic dosing. Biodistribution of TfRMAb-TNFR following a single 3 mg/kg SQ dose (acute) or four weeks of chronic dosing. Tissue concentrations (pg/mg of tissue) were measured in (**A**) kidney, (**B**) liver, (**C**) spleen, and (**D**) brain. Data are shown as the mean ± SEM of *n* = 4–5 mice per group. * *p* < 0.05; ** *p* < 0.01; **** *p* < 0.0001. ns: non-significant.

Following single acute SQ dosing to WT mice, plasma concentrations of TfRMAb-TNFR declined by 97.1% from 310.1 ± 95.1 ng/mL at 3 h to 9.0 ± 4.4 ng/mL at 24 h (Figure 5A,C). In the FcRn KO mice, plasma concentrations of TfRMAb-TNFR declined from 37.4 ± 14.6 ng/mL at 3 h to 3.1 ± 0.0 ng/mL at 24 h (Figure 5A,C). The plasma AUC from 0 to 24 h following a single SQ dose of TfRMAb-TNFR was significantly lower in FcRn KO mice than in WT mice by 88.4% (*p* < 0.05) (Figure 5D). Tissue biodistribution analysis following acute dosing revealed that the concentration of TfRMAb-TNFR in the FcRn KO mice was significantly lower in the kidney, liver, spleen, and brain compared with WT mice by 86% (*p* < 0.05), 80% (*p* < 0.0001), 80% (*p* < 0.05), and 80% (*p* < 0.0001), respectively (Figure 6A–D).

Following four weeks of TfRMAb-TNFR chronic dosing with a 3 mg/kg SQ dose, mice were injected with a final 3 mg/kg SQ dose of TfRMAb-TNFR, and plasma concentrations were measured at 3, 6, and 24 h after this terminal injection. In the WT mice, the plasma concentrations of TfRMAb-TNFR ranged from 3.05 ± 0 ng/mL at 3 h to 7.89 ± 0.76 ng/mL at 24 h (Figure 5B,C). In the FcRn KO mice, plasma concentrations showed similar trends of 3.6 ± 0.58 ng/mL at 3 h to 4.9 ± 0.83 ng/mL at 24 h (Figure 5B,C). There was no significant difference between the plasma concentrations of TfRMAb-TNFR in WT mice and FcRn KO mice after the last injection following chronic dosing (Figure 5B,C). The plasma AUC from 0 to 24 h following the last SQ dose of TfRMAb-TNFR did not differ significantly between the FcRn KO mice and WT mice (Figure 5D). No difference was observed in TfRMAb-TNFR levels in the brain, spleen, or liver between chronically dosed WT and FcRn KO mice. In the kidney, however, TfRMAb-TNFR levels were significantly higher in the FcRn KO mice, showing a 90.3% higher TfRMAb-TNFR concentration compared with WT mice (*p* < 0.05) (Figure 6A–D).

A comparison of acute and chronic dosing showed that chronic dosing resulted in a significant reduction in the plasma exposure of TfRMAb-TNFR compared with a single injection in the WT mice, but there was no difference in plasma exposure between acutely dosed and chronically dosed FcRn KO mice (Figure 5C,D). The plasma AUC following chronic dosing of TfRMAb-TNFR resulted in a 92% reduction (*p* < 0.05) in plasma AUC values in WT mice, with no significant reduction in FcRn KO mice compared with their acute-dosed counterparts (Figure 5D). With respect to tissue biodistribution, paralleling a reduction in plasma concentrations in the WT mice, chronic dosing resulted in a significant decrease in TfRMAb-TNFR tissue levels in the kidney (*p* < 0.01), liver (*p* < 0.0001), spleen (*p* < 0.05), and brain (*p* < 0.0001) compared with concentrations following acute dosing of WT mice (Figure 6A–D). In FcRn KO mice, TfRMAb-TNFR kidney levels were significantly higher following chronic dosing compared with acute dosing (*p* < 0.05) (Figure 6A). On the other hand, TfRMAb-TNFR brain levels were significantly reduced by 94% (*p* < 0.05) in chronically dosed FcRn mice compared with acutely dosed FcRn KO mice (Figure 6D). TfRMAb-TNFR levels remain unchanged in the liver (Figure 6B) and spleen (Figure 6C) of FcRn KO mice following chronic dosing compared with acute dosing.

### 3.4. FcRn Binding of TfRMAb Fusion Proteins and Mouse IgG

The FcRn binding affinities (KD) of chimeric TfRMAb, TfRMAb-EPO, TfRMAb-TNFR, and mouse IgG1 were quantified using an MSD electrochemiluminescence assay. KD for FcRn binding was highest for TfRMAb-TNFR (KD = 114 ± 10.9 nM) compared with TfRMAb-EPO (KD = 7.9 ± 0.07 nM), TfRMAb (KD = 6.7 ± 1.2 nM), and mouse IgG1 (KD = 5.8 ± 0.16 nM) (Table 1).

## 4. Discussion

Previous studies have shown reduced plasma exposure and accelerated clearance following chronic dosing of TfRMAb in mice and non-human primates [17,18,19,21,22]. One possible mechanism of reduced plasma exposure is FcRn saturation [27]. FcRn is essential for maintaining IgG homeostasis, as it binds to the Fc domain of IgG proteins in acidic conditions (pH 6). This interaction occurs at a location distinct from the traditional Fc effector receptor binding site (FcγR) [36]. The key residues at the CH2–CH3 domain interface of the Fc region facilitate binding, which ensures the recycling and extended half-life of IgG molecules, thus enhancing their stability and systemic availability [27,37]. This study focuses on understanding the contribution of FcRn in maintaining plasma pharmacokinetics and tissue biodistribution of identical doses of TfRMAb and TfRMAb fusion proteins in C57BL6 WT mice and FcRn KO mice following acute and chronic administration.

Acute dosing studies showed significantly lower plasma concentrations and/or plasma AUC values in FcRn KO mice for TfRMAb and TfRMAb fusion proteins when compared with WT mice. Interestingly, TfRMAb-TNFR-treated FcRn KO mice demonstrated the most substantial reduction in AUC (~90%). Similarly, TfRMAb and TfRMAb-EPO cleared from the plasma of FcRn KO mice more quickly than from WT mice within 24 h, although the impact on overall plasma AUC was less compared with that for TfRMAb-TNFR. These observations align with previous reports that FcRn plays a critical role in regulating IgG half-life by protecting it from lysosomal degradation through pH-dependent binding and recycling or transcytosis [28,37]. For instance, engineered IgG subclasses or Fc fusion proteins with enhanced FcRn binding at acidic pH (pH 6.0) have prolonged half-life and improved therapeutic efficacy [38]. Furthermore, we observe that the associated fusion partner differentially affects the contribution of FcRn recycling in regulating IgG persistence, which may be linked to their interaction with FcRn. Accordingly, previous findings demonstrate that Fc fusion proteins bind to FcRn less effectively than IgG, possibly due to steric hindrance or structural changes that may interfere with FcRn interaction [7].

IgG recycling by FcRn can be saturable [27], and studies have shown that chronic dosing can saturate FcRn and increase IgG catabolism [39,40]. In our hands, the plasma pharmacokinetics of the TfRMAb fusion proteins following chronic dosing demonstrated varying degrees of dependence on FcRn-mediated recycling. Compared with acute dosing, plasma exposure of TfRMAb and TfRMAb-EPO declined in the FcRn KO mice following chronic dosing. Therefore, despite the absence of FcRn, chronic dosing resulted in reduced plasma exposure of TfRMAb and TfRMAb-EPO even in FcRn KO mice, indicating that additional mechanisms beyond FcRn saturation largely regulate their plasma concentrations following chronic dosing. In contrast, in TfRMAb-TNFR-treated mice, only WT mice showed a decline in plasma concentrations following chronic dosing in comparison with acute dosing, while plasma concentrations in FcRn KO mice were relatively unchanged between acute and chronic dosing. Furthermore, no significant changes were observed in the plasma concentrations of TfRMAb-TNFR between WT and FcRn KO mice at 3, 6, and 24 h during chronic dosing, suggesting that FcRn saturation may be implicated. Comparing these profiles, we can likely deduce that even though there may be changes in FcRn recycling due to continuous antibody exposure via chronic dosing, there are likely to be other mechanisms that contribute to the retention or clearance of these fusion proteins following chronic dosing. Studies show that factors beyond FcRn recycling may significantly affect the clearance and pharmacokinetics of TfRMAb therapeutics following chronic dosing [17,21,22]. In this regard, the formation of ADA has been implicated in reduced plasma exposure of TfRMAb fusion proteins following chronic administration [21,22]. Other mechanisms that have been suggested include increased expression of TfR by peripheral organs [17], which can increase TfRMAb uptake from the blood circulation and reduce plasma exposure. Additionally, sequestration of TfRMAb fusion proteins by TfR on blood cells has been shown to reduce TfRMAb bioavailability in aged mice [41].

FcRn is essential for managing the distribution and availability of IgGs and Fc fusion proteins in the body [27], and its tissue expression levels differ among different organs [42]. FcRn enables the bidirectional transport of IgG across mucosal surfaces and epithelial membranes, efficiently recycling antibodies and ensuring their systemic availability [42]. Studies show that FcRn transcytosis may play a role in the transport of antibodies between tissues and peripheral circulation, highlighted by the differences in tissue bioavailability of antibodies observed in FcRn KO and WT models [42]. In the current study, acutely dosed WT mice consistently exhibited greater tissue biodistribution across all TfRMAb constructs and organs, including kidney, liver, spleen, and brain, compared with acutely dosed FcRn KO mice and chronically dosed mice. This observation likely indicates that tissue biodistribution is driven by the plasma exposure of the TfRMAb constructs. However, a closer look at the tissue-to-plasma ratio showed that FcRn also regulates tissue biodistribution of the TfRMAb constructs. For example, despite the higher plasma concentrations of TfRMAb in WT compared with FcRn KO mice following acute dosing, TfRMAb showed higher tissue-to-plasma ratios in the kidney, spleen, and brain (with the same trend in the liver) in FcRn KO acute-dosed mice (Supplemental Figure S2). An increase in the tissue-to-plasma ratio of TfRMAb in the FcRn KO mice suggests that FcRn plays an important role in tissue efflux in these organs. This finding is consistent with existing literature indicating that FcRn-mediated transcytosis aids in the transport of antibodies between the bloodstream and tissues [42,43] and that elevated IgG1 levels are found in FcRn KO mice at later time points, suggesting a role of FcRn in IgG efflux from tissues [42]. This is also consistent with previous work showing that the BBB FcRn regulates the efflux of IgG from the brain parenchyma [43]. Notably, this increase in tissue-to-plasma ratio was not seen in the presence of a fusion partner (Supplemental Figure S2), suggesting that the fusion partner could possibly influence FcRn binding dynamics and, in turn, affect tissue biodistribution. However, there was one exception. FcRn KO mice chronically dosed with TfRMAb-TNFR showed increased kidney accumulation, which is consistent with the role of FcRn in tissue efflux and with reports showing that mice lacking FcRn accumulate IgG in the glomerular basement membrane [44]. This effect is more pronounced for TfRMAb-TNFR than for the other fusion proteins, likely due to its distinct FcRn binding (Table 1), which plays a major role in shaping its pharmacokinetic profile. The increased renal accumulation of TfRMAb-TNFR in FcRn KO mice was evident only after chronic dosing, and not after acute dosing, most likely due to cumulative tissue TfRMAb-TNFR uptake.

Hematologic and metabolic panel indices provide a comprehensive evaluation of systemic health, facilitating the assessment of TfRMAb fusion proteins by tracking key biomarkers related to organ function, metabolic stability, and potential systemic effects, ensuring both efficacy and safety in development and application. Following acute dosing, both treated and untreated FcRn KO mice exhibited lower albumin levels compared with fusion protein-treated and untreated WT mice (Supplemental Figure S3), consistent with impaired FcRn-mediated albumin recycling, as FcRn is essential for this process [45]. Reduced calcium and total protein levels in both treated and untreated FcRn KO mice, compared with the WT mice, are likely a consequence of hypoalbuminemia [46]. The higher globulin levels observed in FcRn KO mice compared with their WT counterparts (treated or untreated) may reflect a compensatory feedback mechanism for reduced albumin [47]. These shifts in metabolic indices occur in both treated and untreated FcRn KO mice, indicating that they arise from FcRn deficiency itself rather than from TfRMAb fusion protein treatment.

Hematologic indices of WT and FcRn KO mice after chronic treatment of fusion proteins were compared with untreated WT or untreated FcRn KO mice (Supplemental Figure S4). Chronic TfRMAb treatment was associated with splenomegaly (Supplemental Figure S1) and hematologic changes, including lower RBC, hematocrit, and hemoglobin compared with control values (Supplemental Figure S4) in WT and FcRn KO mice, indicating that these changes are driven by TfRMAb and not FcRn. This finding is consistent with previous studies, which have shown a reduction in reticulocytes associated with TfRMAb treatment [33,48]. As for TfRMAb-EPO-treated or TfRMAb-TNFR-treated WT and FcRn KO mice, there were no changes to the RBC, hematocrit, and hemoglobin compared with their respective controls. Splenomegaly was not observed with TfRMAb-EPO and TfRMAb-TNFR treatment. These varying effects observed with TfRMAb, TfRMAb-EPO, and TfRMAb-TNFR treatment again emphasize the role of the fusion partner in regulating the side effects of TfRMAb constructs [17].

The different pharmacokinetic profiles of TfRMAb-TNFR, TfRMAb, and TfRMAb-EPO in FcRn KO mice likely result from differences in FcRn binding, which regulates IgG recycling and half-life. The binding affinity of TfRMAb-TNFR for FcRn was 14- to 16-fold lower than that of TfRMAb and TfRMAb-EPO (Table 1); this could imply superior FcRn-mediated recycling of the latter two constructs and a greater impact of FcRn in regulating their pharmacokinetics. However, our work shows that TfRMAb-TNFR pharmacokinetics were the most impacted by the absence of FcRn. Studies have demonstrated that strong FcRn binding may impair exocytosis into the blood circulation and fail to improve IgG half-life, affecting IgG pharmacokinetics and persistence [49,50,51]. Therefore, it is conceivable that the stronger FcRn affinity of TfRMAb and TfRMAb-EPO hinders IgG exocytosis into the blood circulation, offsetting the benefits of FcRn recycling. This may explain the reduced reliance of TfRMAb and TfRMAb-EPO on FcRn for plasma retention. In contrast, the weaker FcRn binding of TfRMAb-TNFR may enhance exocytosis into the bloodstream and increase FcRn-mediated protection, an effect absent in FcRn KO mice, thereby explaining its greater dependence on FcRn recycling.

## 5. Conclusions

In conclusion, this study demonstrates the differential role of FcRn in regulating the pharmacokinetics and tissue biodistribution of TfRMAb therapeutic proteins. Acute dosing revealed the significant contribution of FcRn to maintaining plasma levels, while chronic dosing showed some evidence of potential saturation of FcRn recycling pathways and a greater likelihood of involvement of alternative, non-FcRn mechanisms, in influencing plasma pharmacokinetics. Moreover, fusion partners significantly influenced how FcRn-mediated recycling shaped the pharmacokinetics of TfRMAb fusion proteins. These findings highlight the challenge of establishing a universal framework, such as modulating FcRn binding affinity for optimizing the pharmacokinetics of TfRMAbs, underscoring the need for individualized assessment in each case. For instance, in situations where FcRn recycling does not significantly influence plasma bioavailability, Fab fusion proteins lacking Fc effector functions may be engineered to potentially reduce side effects associated with Fc domain interactions.

## Figures and Tables

**Table 1:** FcRn binding of mouse IgG1, chimeric TfRMAb, and TfRMAb fusion proteins. KD: equilibrium dissociation constant (mean ± SEM).

Fusion Protein	KD (nM)
TfRMAb-TNFR	114 ± 10.9
Mouse IgG1	5.8 ± 0.16
TfRMAb-EPO	7.9 ± 0.07
TfRMAb	6.7 ± 1.2

## Data Availability

The original contributions presented in this study are included in the article/Supplementary Materials. Further inquiries can be directed to the corresponding authors.

## Supplementary Materials

The following supporting information can be downloaded at: https://www.mdpi.com/article/10.3390/pharmaceutics18020269/s1, Figure S1. Organ weights following acute and chronic dosing of TfRMAb-EPO, TfRMAb, and TfRMAb-TNFR. Figure S2. Tissue-to-plasma ratio of TfRMAb-EPO, TfRMAb, and TfRMAb-TNFR following acute and chronic dosing. Figure S3. Biochemical parameters in TfRMAb-EPO-, TfRMAb-, and TfRMAb-TNFR-treated mice following acute dosing. Figure S4. Hematologic parameters in TfRMAb-EPO-, TfRMAb-, and TfRMAb-TNFR-treated mice following chronic dosing.

## Abbreviations

The following abbreviations are used in this manuscript:

AD	Alzheimer’s disease
TfRMAbs	Transferrin receptor-targeting monoclonal antibodies
FcRn	Neonatal Fc receptor
WT	Wild-type
SQ	Subcutaneous
RMT	Receptor-mediated transcytosis
ADA	Anti-drug antibody
PK	Pharmacokinetics
BBB	Blood–brain barrier
TfR	Transferrin receptor-1
PBS	Phosphate-buffered saline
MSD	Mesoscale discovery
BSA	Bovine serum albumin
