# Supplementary material for: Impact of Neonatal Fc Receptor on Transferrin Receptor Antibody Fusion Protein Pharmacokinetics
Source: Pharmaceutics. 2026 Feb 22;18(2):269. doi: 10.3390/pharmaceutics18020269 (PMC12944049; doi:10.3390/pharmaceutics18020269)
Supplement: Supplementary file 1 [file pharmaceutics-18-00269-s001.zip › pharmaceutics-4095995-supplementary.pdf]

pharmaceutics

MDPI

Supplementary Materials: Impact of Neonatal Fc Receptor on Transferrin Receptor Antibody Fusion Protein Pharmacokinetics

Adenike Oyegbesan$ [1], Nataraj Jagadeesan$ [1], Devaraj V. Chandrashekar [1] and Rachita K. Sumbria [1,2,3,*]

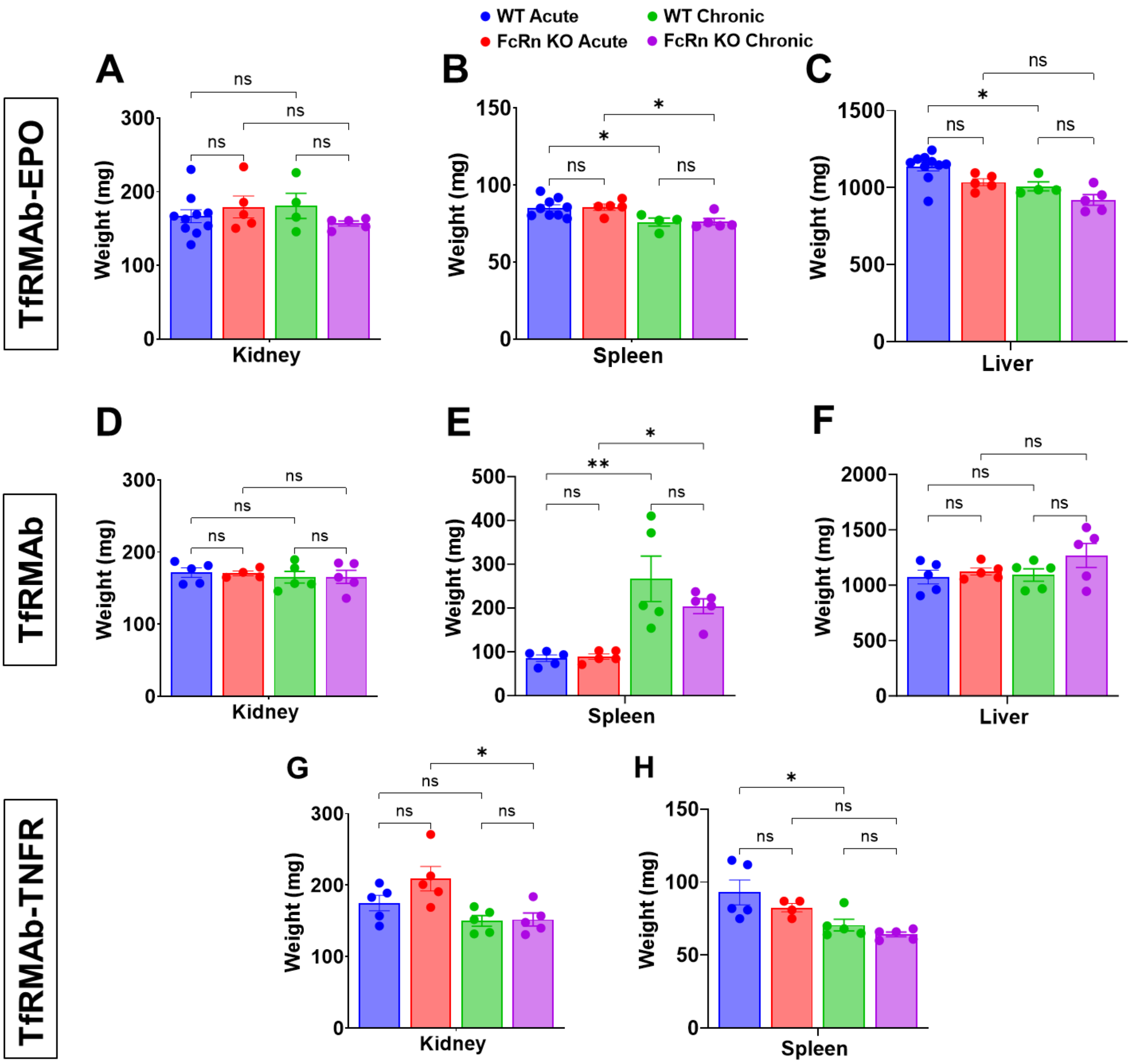

Figure S1. Organ weights following acute and chronic dosing of TfRMAb-EPO, TfRMAb, and TfRMAb-TNFR. WT and FcRn KO mice received acute (3 mg/kg SQ) or chronic (3 mg/kg SQ three days a week for 4 weeks) dosing, and organ weights were assessed for (A, D, G) kidney, (B, E, H) spleen, and (C, F) liver. Livers were not weighed for the TfRMAb-TNFR study. Data are shown as mean ± SEM of n = 4-10 mice per group. * $p < 0.05$, ** $p < 0.01$, ns: non-significant.

 https://doi.org/10.3390/xxxxx

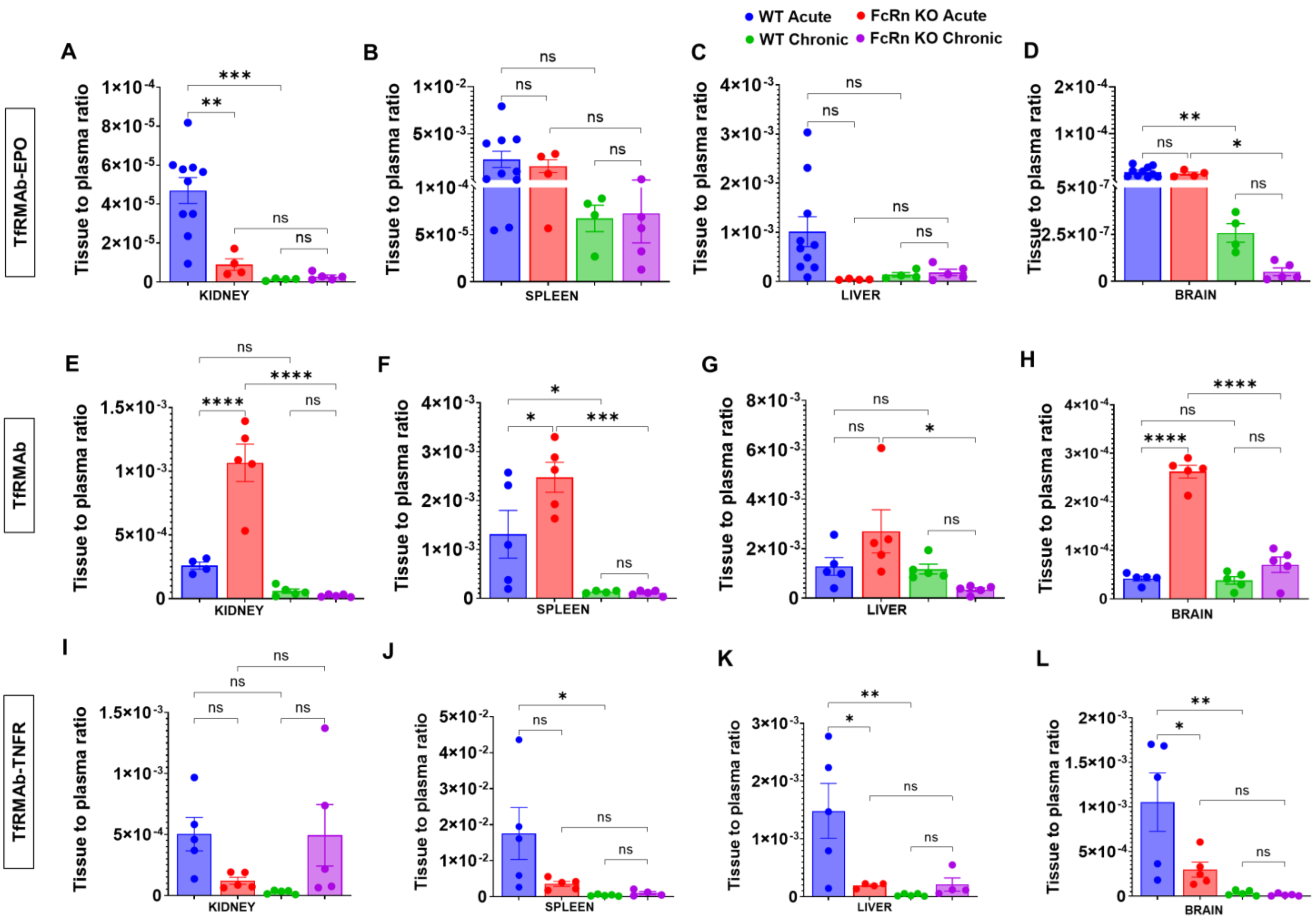

Figure S2. Tissue-to-plasma ratio of TfRMAb-EPO, TfRMAb, and TfRMAb-TNFR following acute and chronic dosing. Tissue-to-plasma ratio in the (A, E, I) kidney, (B, F, J) spleen, (C, G, K) liver, and (D, H, L) brain was measured in WT and FcRn KO mice following acute and chronic dosing. The tissue-to-plasma ratio is an approximation for acute TfRMAb-TNFR data, because the below-the-detection plasma values were replaced with the lowest detectable concentration of the assay. Data are shown as mean ± SEM of n = 4-10 mice per group. *p < 0.05, **p < 0.01, ***p < 0.001, ****p < 0.0001, ns: non-significant.

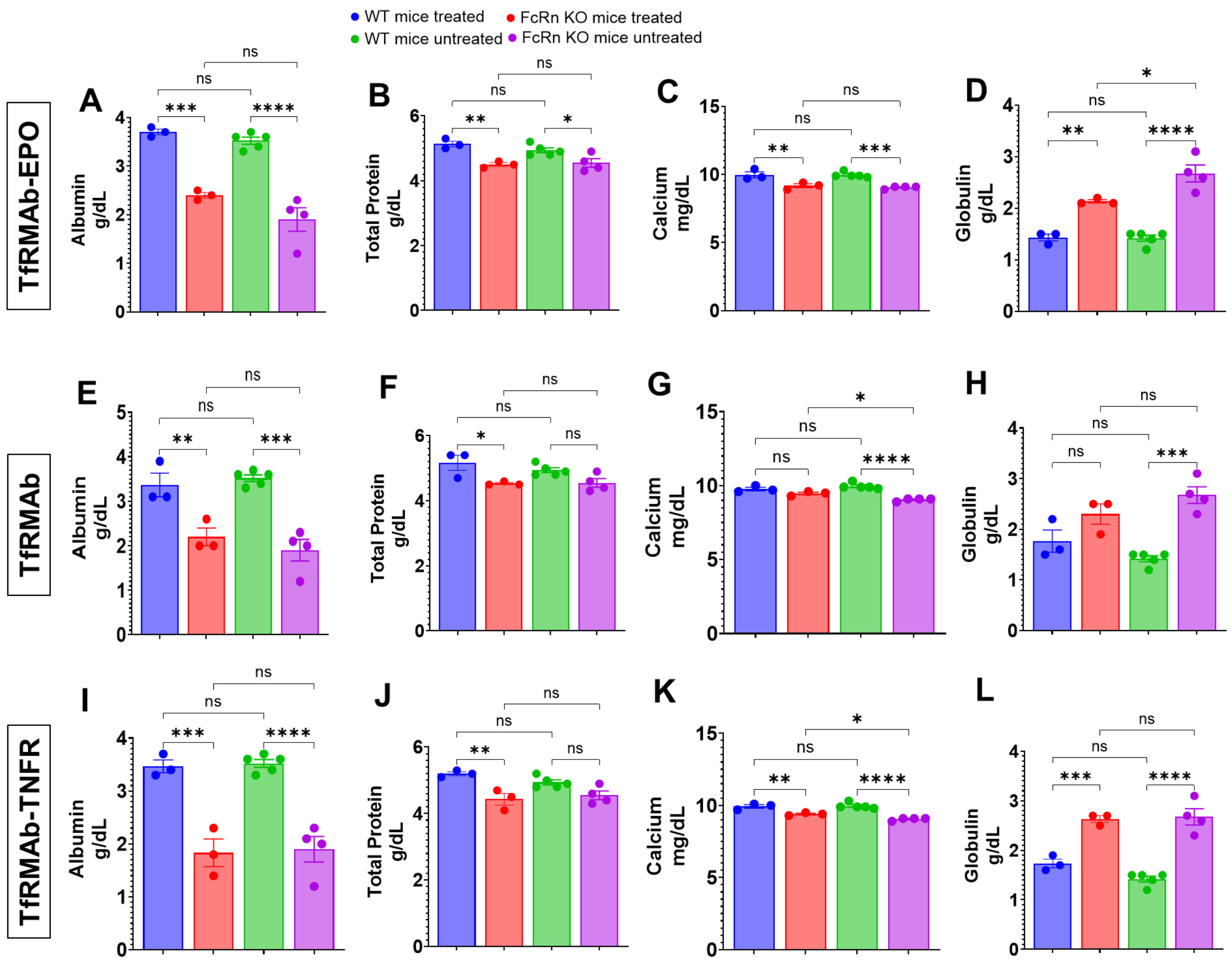

Figure S3. Biochemical parameters in TfRMAb-EPO-, TfRMAb-, and TfRMAb-TNFR-treated mice following acute dosing. Plasma levels of (A, E, I) albumin, (B, F, J) total protein, (C, G, K) calcium, and (D, H, L) globulin were measured in WT and FcRn KO mice. Data are shown as mean ± SEM of n = 3-4 mice per group. $^{*}p < 0.05$, $^{**}p < 0.01$, $^{***}p < 0.001$, $^{****}p < 0.0001$, ns: non-significant. Note: the untreated mice are the same across all groups.

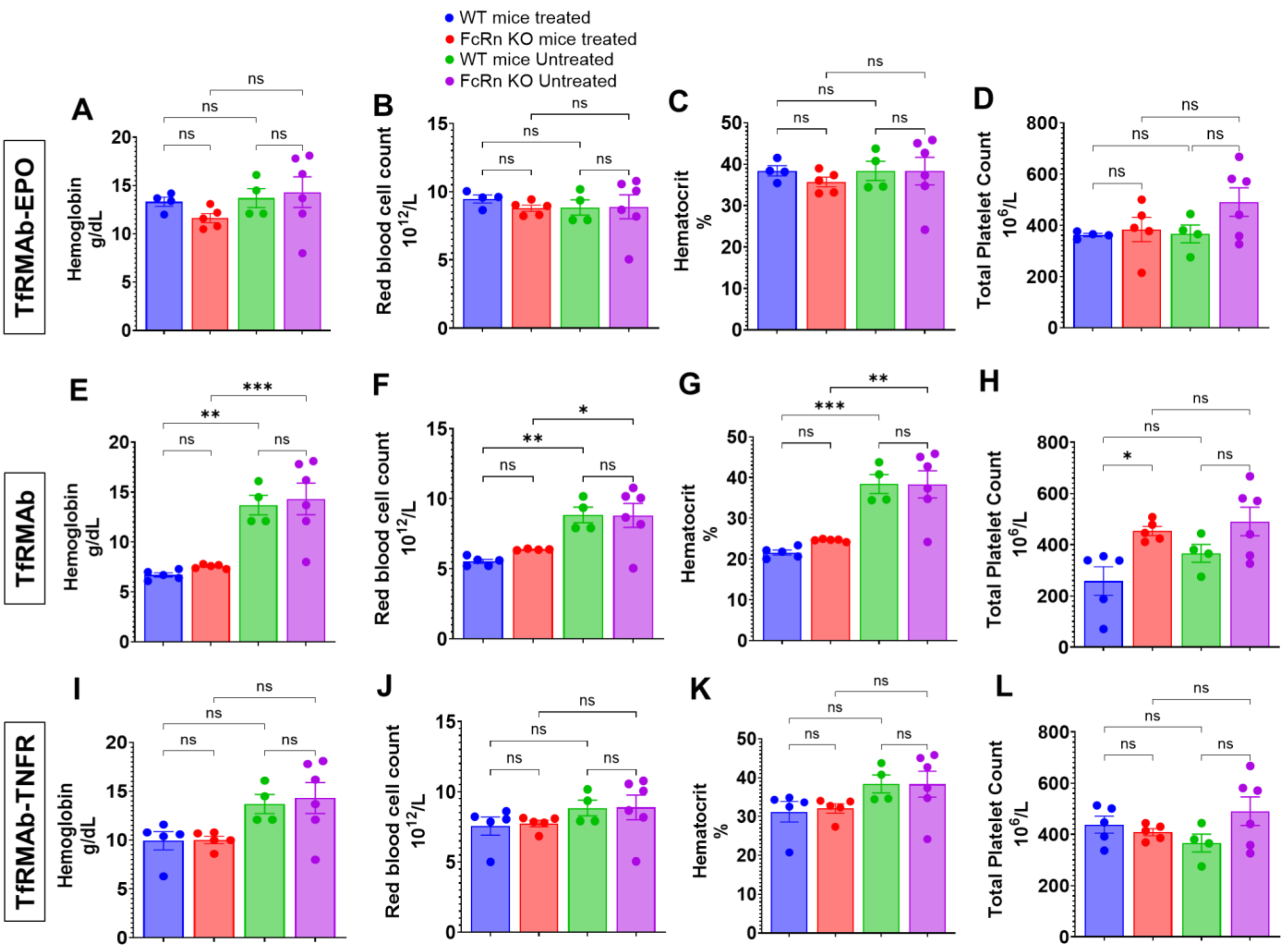

Figure S4. Hematologic parameters in TfRMAb-EPO-, TfRMAb-, and TfRMAb-TNFR-treated mice following chronic dosing. Plasma levels of (A, E, I) hemoglobin, (B, F, J) red blood cell count, (C, G, K) hematocrit, and (D, H, L) platelet count were measured in WT and FcRn KO mice. Data are shown as mean ± SEM of n = 4–6 mice per group. *p < 0.05, **p < 0.01, ***p < 0.001, ns: non-significant. Note: the untreated mice are the same across all groups.
